# Supplementary material for: Rational Fabrication of Defect-Rich and Hierarchically Porous Fe-N-C Nanosheets as Highly Efficient Oxygen Reduction Electrocatalysts for Zinc-Air Battery
Source: Molecules. 2023 Mar 23;28(7):2879. doi: 10.3390/molecules28072879 (PMC10095661; doi:10.3390/molecules28072879)
Supplement: Supplementary file 1 [file molecules-28-02879-s001.zip › molecules-2269454-supplementary.pdf]

Supporting Information

Rational Fabrication of Defect-Rich and Hierarchically Porous Fe-N-C Nanosheets as Highly Efficient Oxygen Reduction Electrocatalysts for Zinc-Air Battery

Sensen Li, Yan Lv, Sawida Elam, Xiuli Zhang, Zhuojun Yang, Xueyan Wu * and Jixi Guo *

State Key Laboratory of Chemistry and Utilization of Carbon Based Energy Resources, College of Chemistry, Xinjiang University, Urumqi 830017, China
* Correspondence: wuxy90@xju.edu.cn (X.W.); jxguo1012@163.com (J.G.)

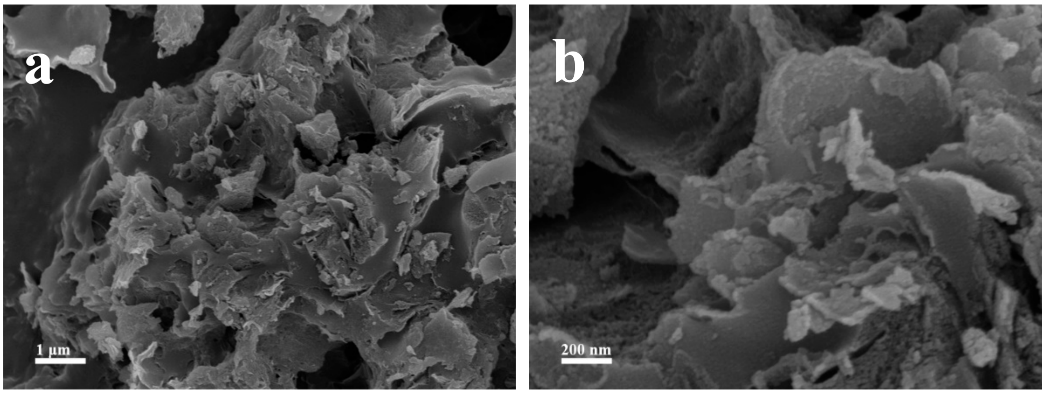

Figure S1. SEM images of Fe-N-CNSs-800.

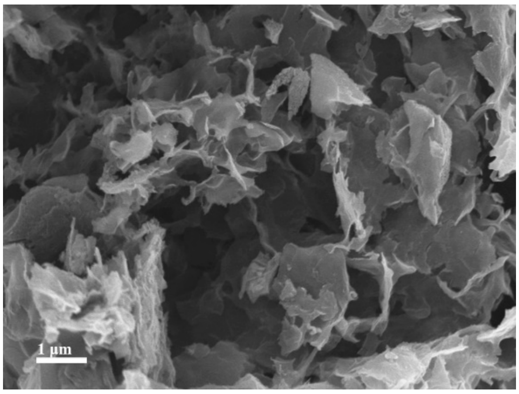

Figure S2. SEM image of Fe-N-CNSs-900.

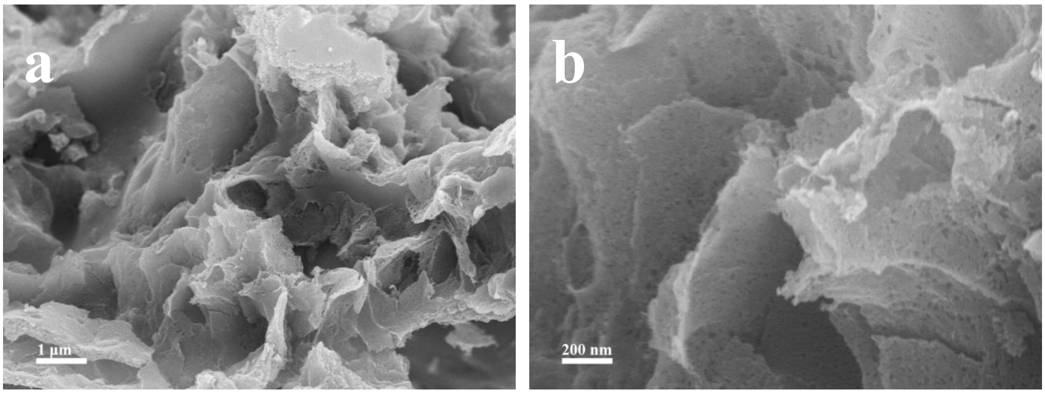

Figure S3. SEM images of Fe-N-CNSs-1000

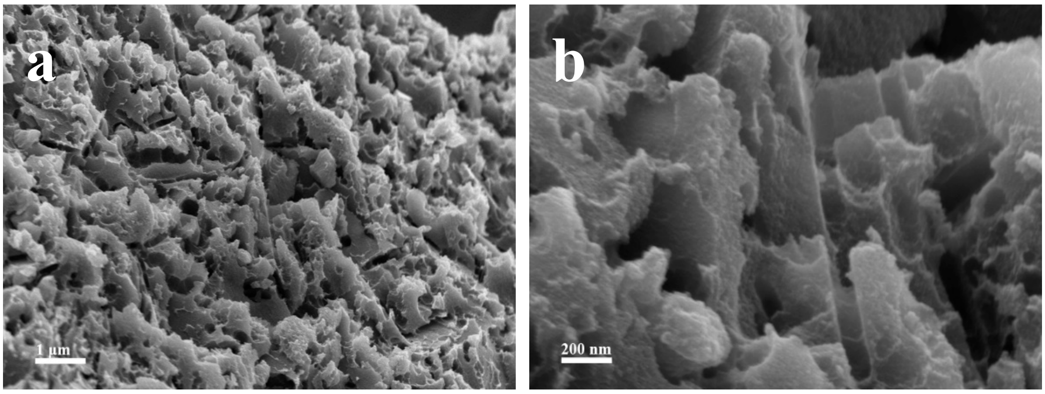

Figure S4. SEM images of Fe-N-C-900.

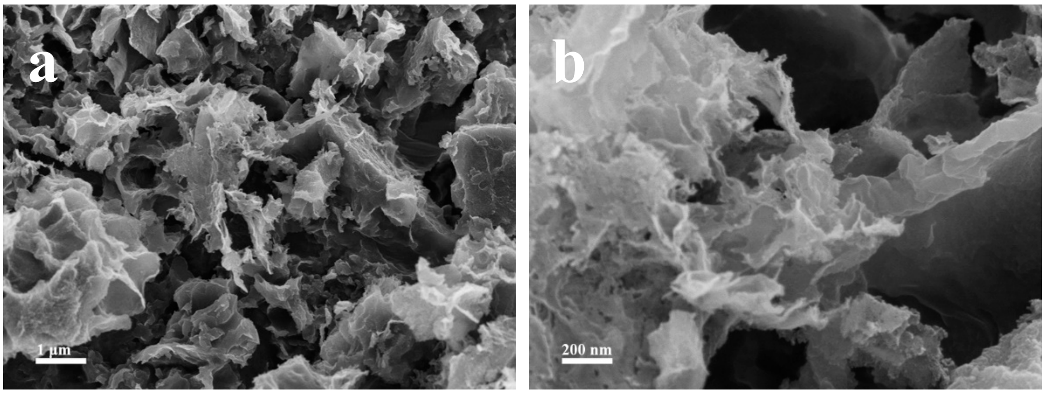

Figure S5. SEM images of Fe-N-CNSs-15.

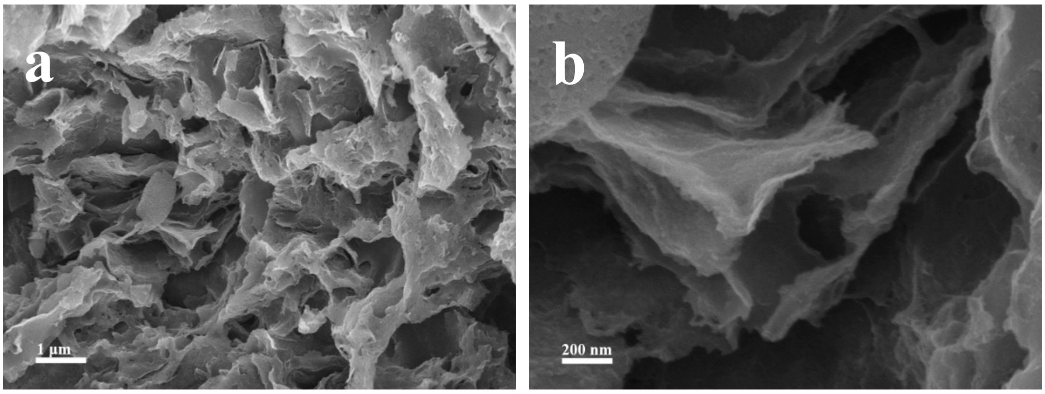

Figure S6. SEM images of Fe-N-CNSs-60.

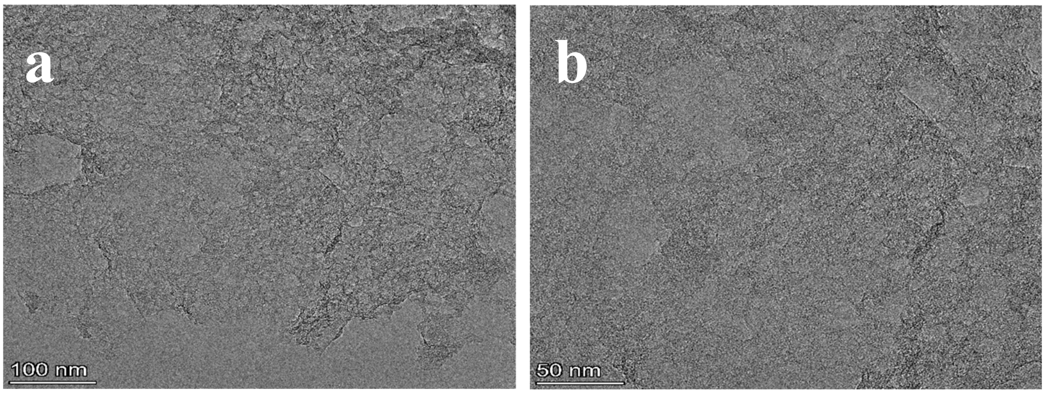

Figure S7. TEM images of Fe-N-CNSs.

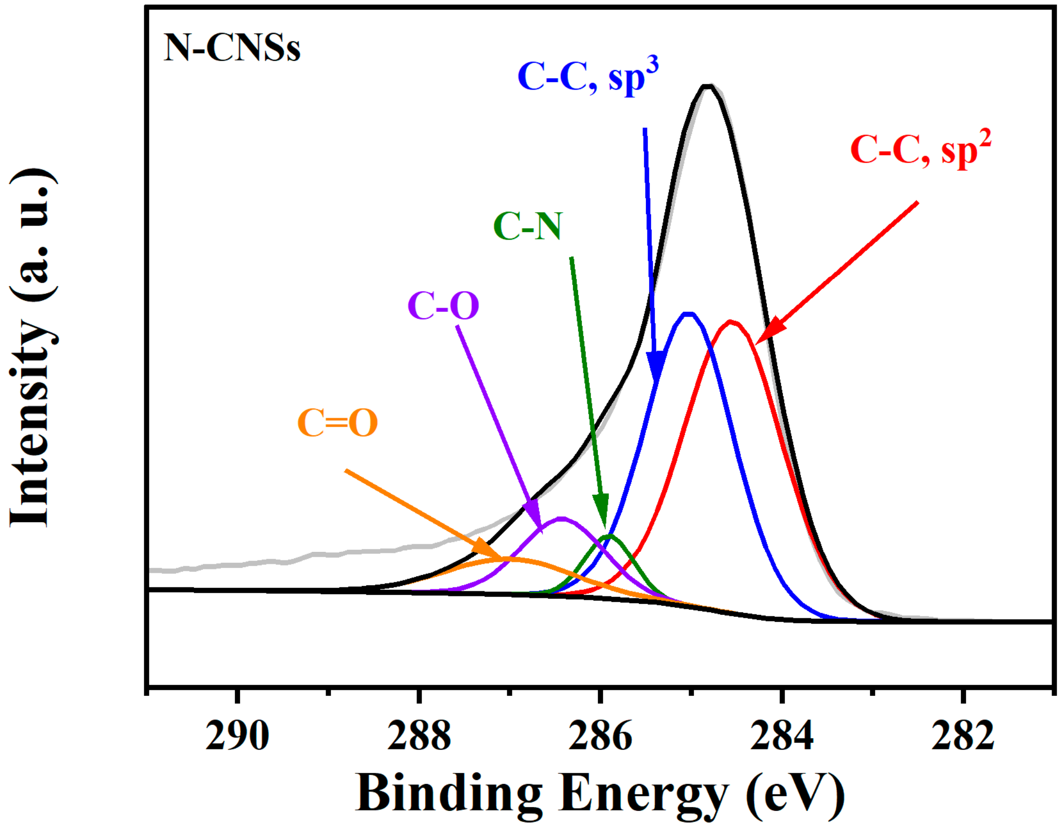

Figure S8. High-resolution C 1s spectrum of N-CNSs.

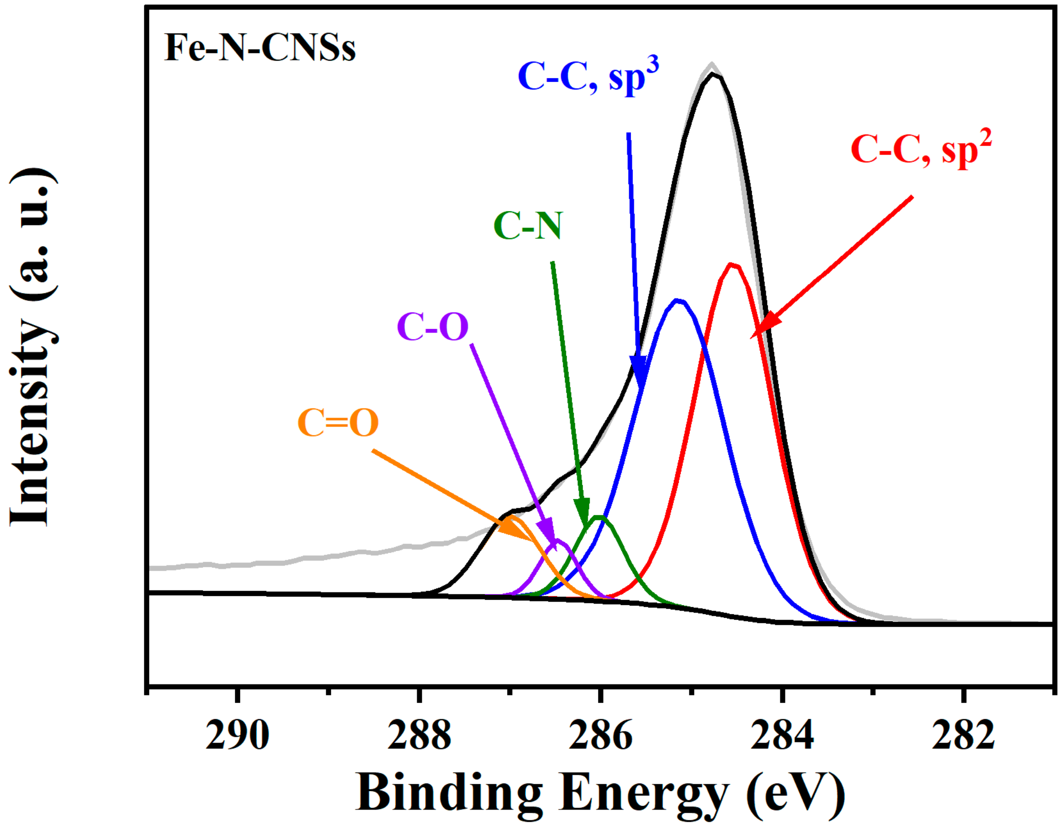

Figure S9. High-resolution C 1s spectrum of Fe-N-CNSs.

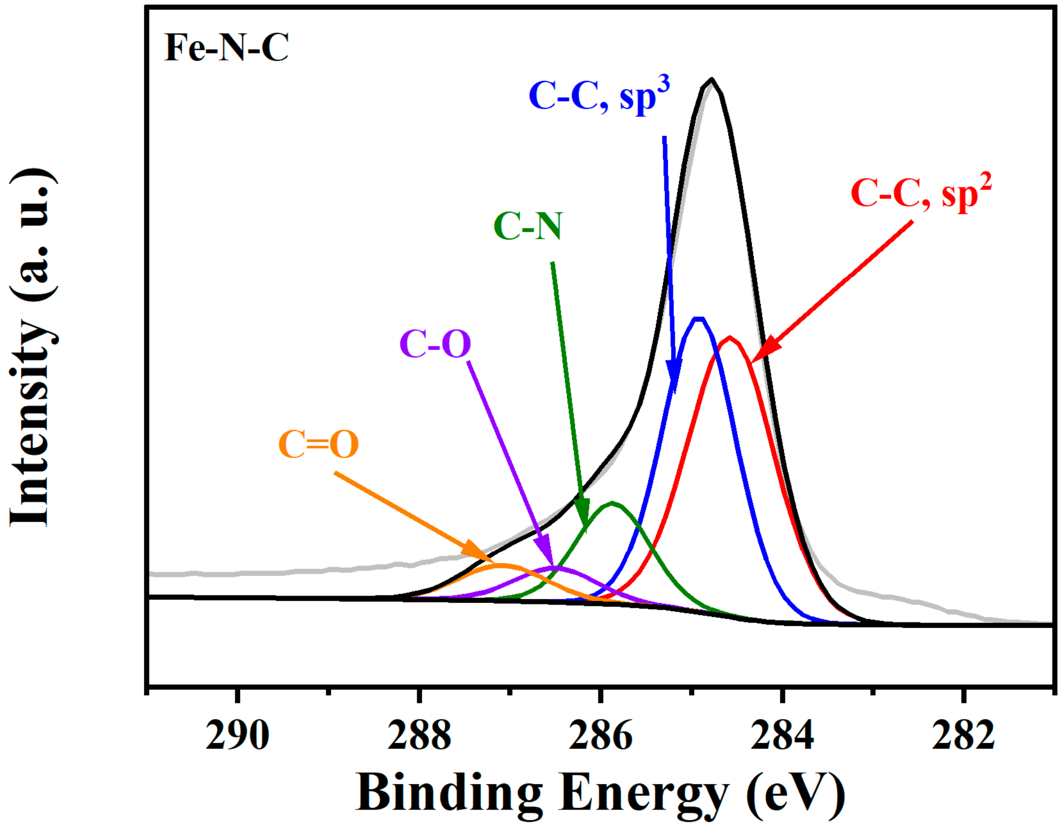

Figure S10. High-resolution C 1s spectrum of Fe-N-C.

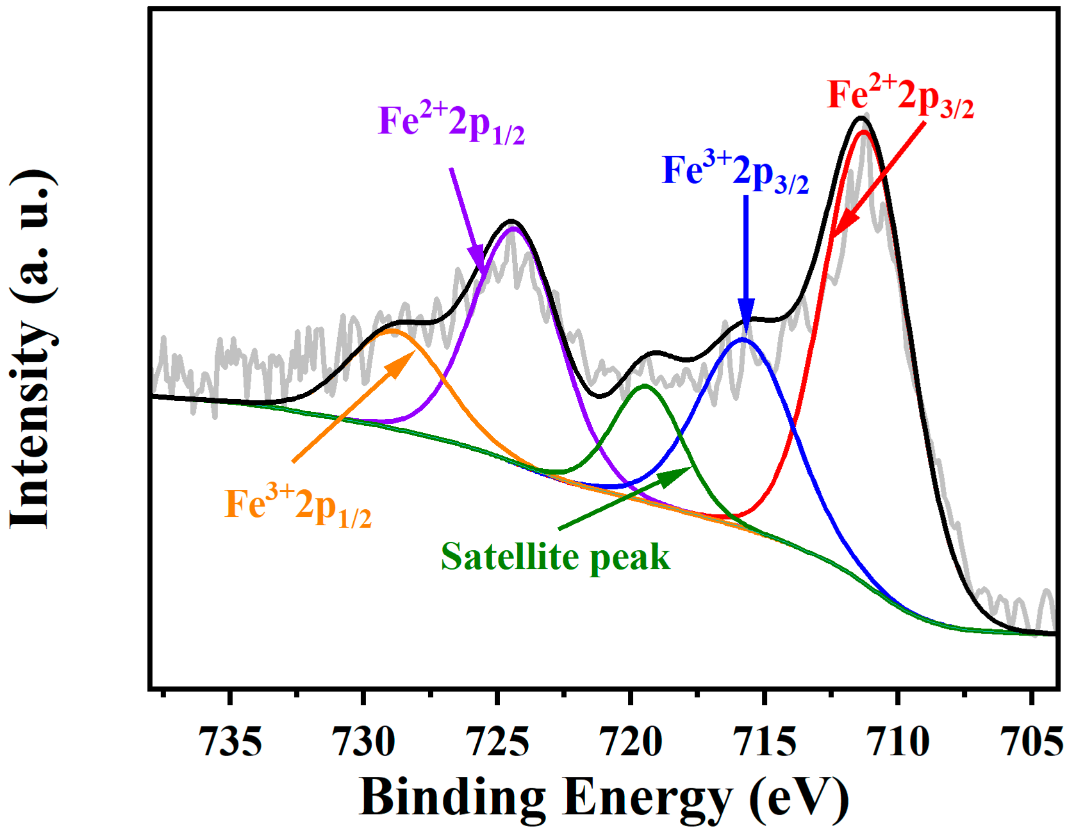

Figure S11. High-resolution Fe 2p spectrum of Fe-N-CNSs.

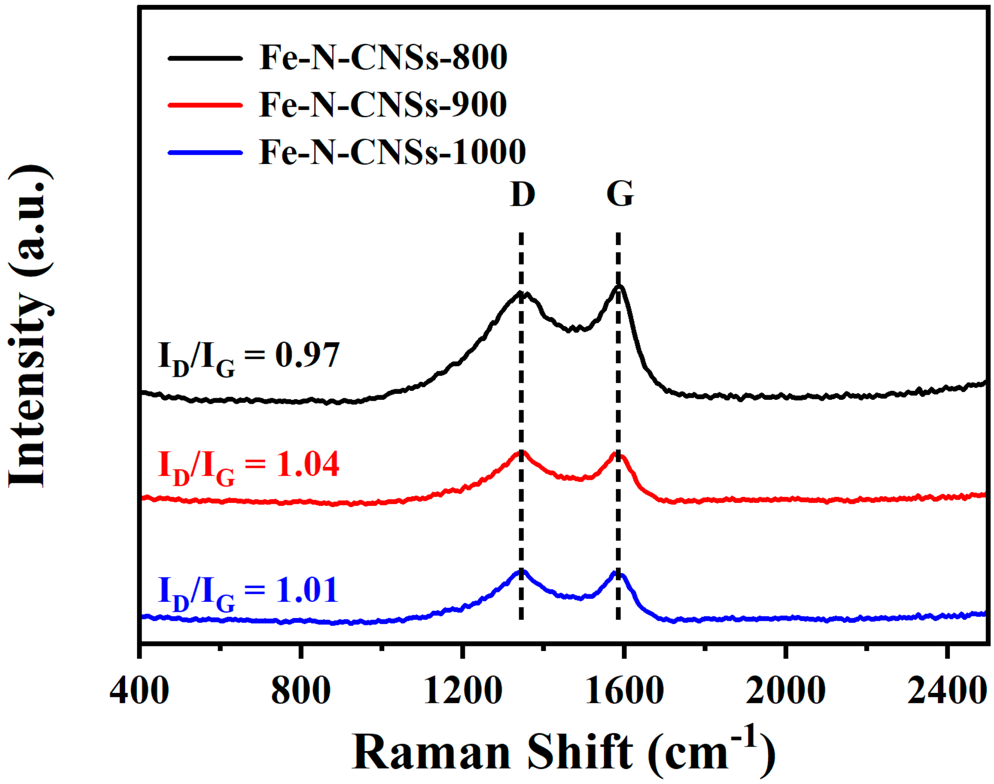

Figure S12. Raman spectra of the synthesized materials.

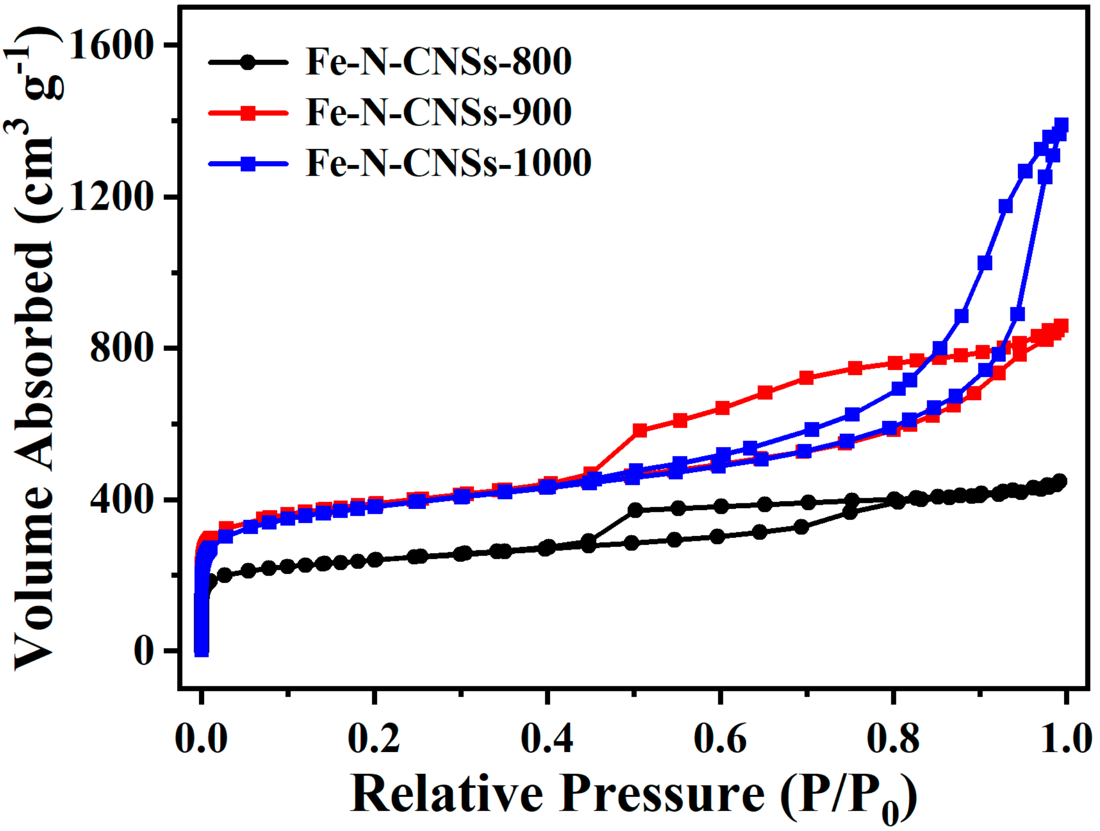

Figure **S13**. N_2 adsorption-desorption isotherms of Fe-N-CNSs-800, Fe-N-CNSs-900 and Fe-N-CNSs-1000.

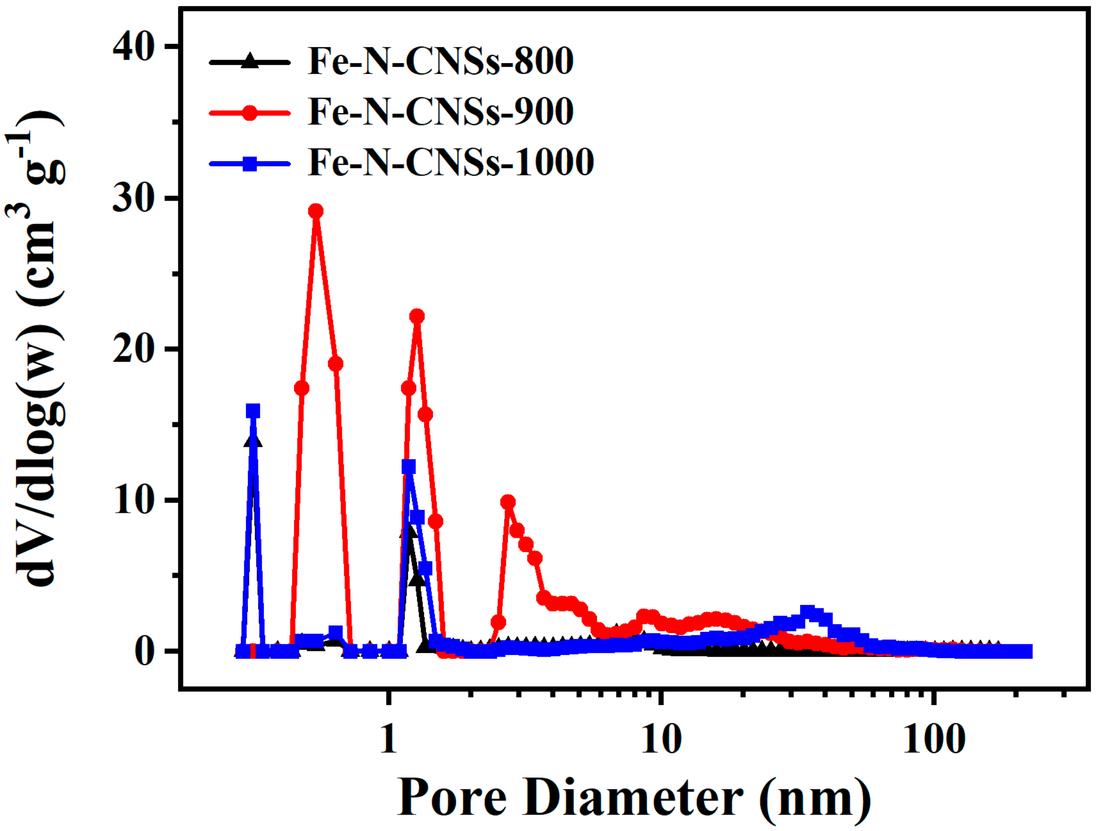

Figure **S14**. The pore size distribution of Fe-N-CNSs-800, Fe-N-CNSs-900 and Fe-N-CNSs-1000.

Table S1. Comparison of ORR activity of various Fe-N-C catalysts.

Catalysts	Electrolyte	$E_{1/2}$(V vs. RHE)	Reference
**Fe-N-CNSs**	**0.1 M KOH**	**0.87**	**This work**
Fe-SAC/NC	0.1 M KOH	0.84	*Nano Energy* **2020,** 72, 104670.
Fe-N-C/NG	0.1 M KOH	0.84	*J. Alloy. Compd.* **2022,** 901, 163763.
Fe-AC-2	0.1 M KOH	0.87	*J. Mater. Chem.* ***A*** **2021,** 9, 7137–7142.
Fe-N-C/HPC-NH_3	0.1 M KOH	0.80	*Nano Res.* **2019,** 12, 1651–1657.
AC-N-Fe	0.1 M KOH	0.76	*Chem. Eng. J.* **2022,** 446, 137256.
Fe-mela-mPD-N-C	0.1 M KOH	0.86	*J. Alloy. Compd.* **2023,** 935, 168069.
FeN4-O-NCR	0.1 M KOH	0.94	*Adv. Mater.* **2022,** 34, 2202544.
Fe-N-C/MXene	0.1 M KOH	0.84	*ACS Nano* **2020,** 14, 2436–2444.
FeNSC-ZM	0.1 M KOH	0.87	*Chem. Eng. J.* **2022,** 444, 136433.

Fe/Meso-NC-1000	0.1 M KOH	0.88	*Adv. Mater.* **2022,** 34, 2107291.
SA Fe@ZrO_2/NC	0.1 M KOH	0.86	*Chem. Eng. J.* **2021,** 420, 129938.

Table S2. Comparison of the performance of the primary zinc-air batteries with the present work.

Catalysis	Peak power density ($mW\ cm^{-2}$)	Reference
**Fe-N-CNSs**	**128.20**	**This work**
IrFe-N-C	113.9	*ACS Catal.* **2022,** 12, 9397-9409
M-Fe-NCNSs	110.5	*Chem. Eng. J.* **2020,** 393, 124702
FeN_x/S-NC	83	*Appl. Surf. Sci.* **2022,** 580, 152255
Fe-N-C	167	*Nano Energy* **2022,** 104, 107869
FeNC	118.05	*Materials Today Energy* **2020,** 18, 100500
AC-N-Fe	124.2	*Chem. Eng. J.* **2022,** 446, 137256
P-Fe-N-CNTs	145	*Appl. Catal. B: Environ.* **2023,** 327, 122469
Fe_3-N-C-800	80	*New J. Chem.* **2022,** 46,989-994

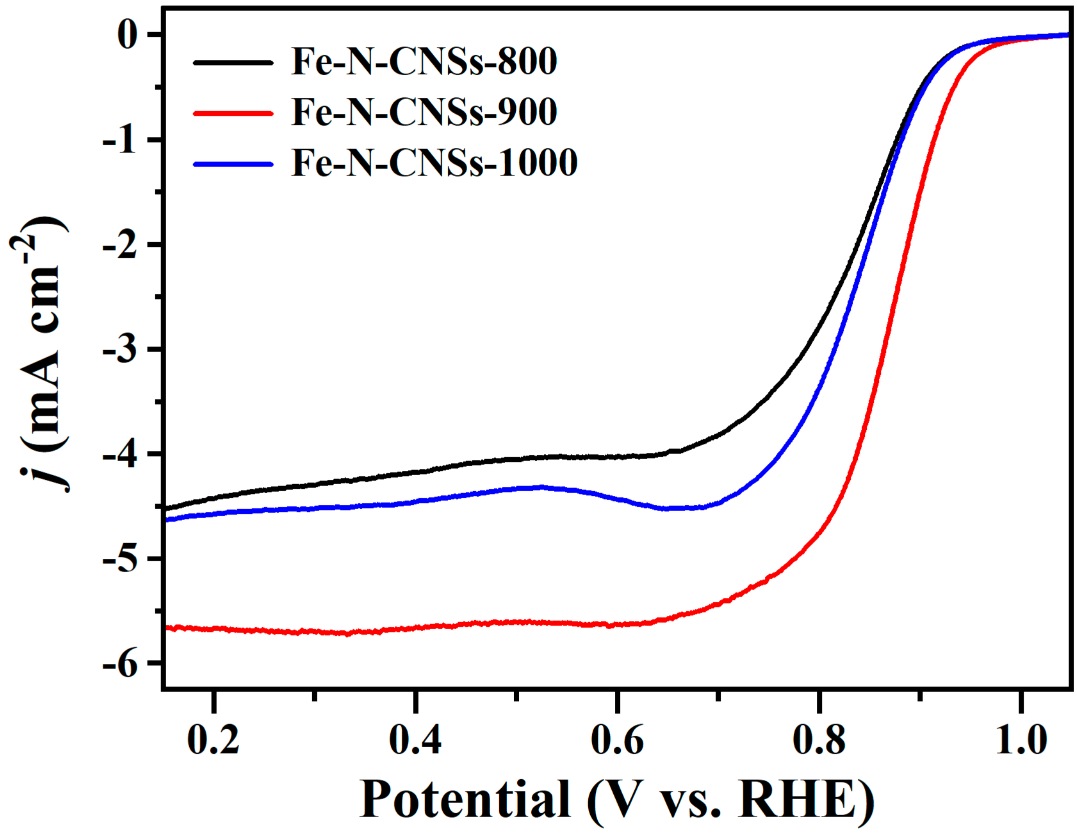

Figure **S15.** LSV curves of Fe-N-CNSs-800, Fe-N-CNSs-900, Fe-N-CNSs-1000.

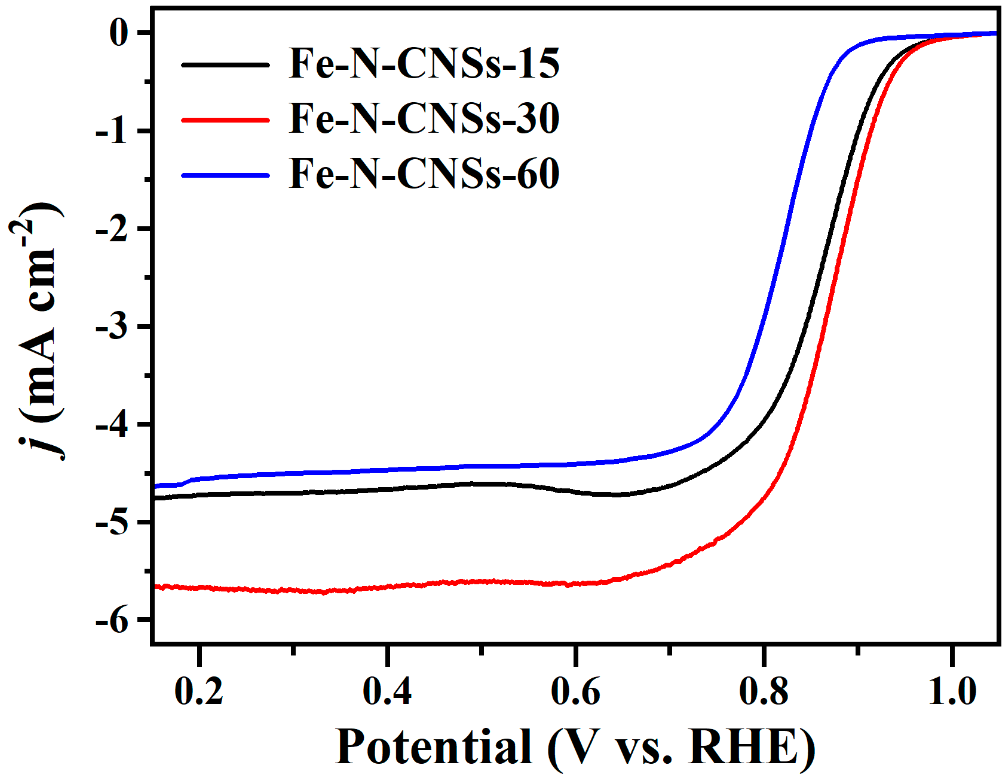

Figure **S16.** LSV curves of Fe-N-CNSs-15, Fe-N-CNSs-30, Fe-N-CNSs-60.

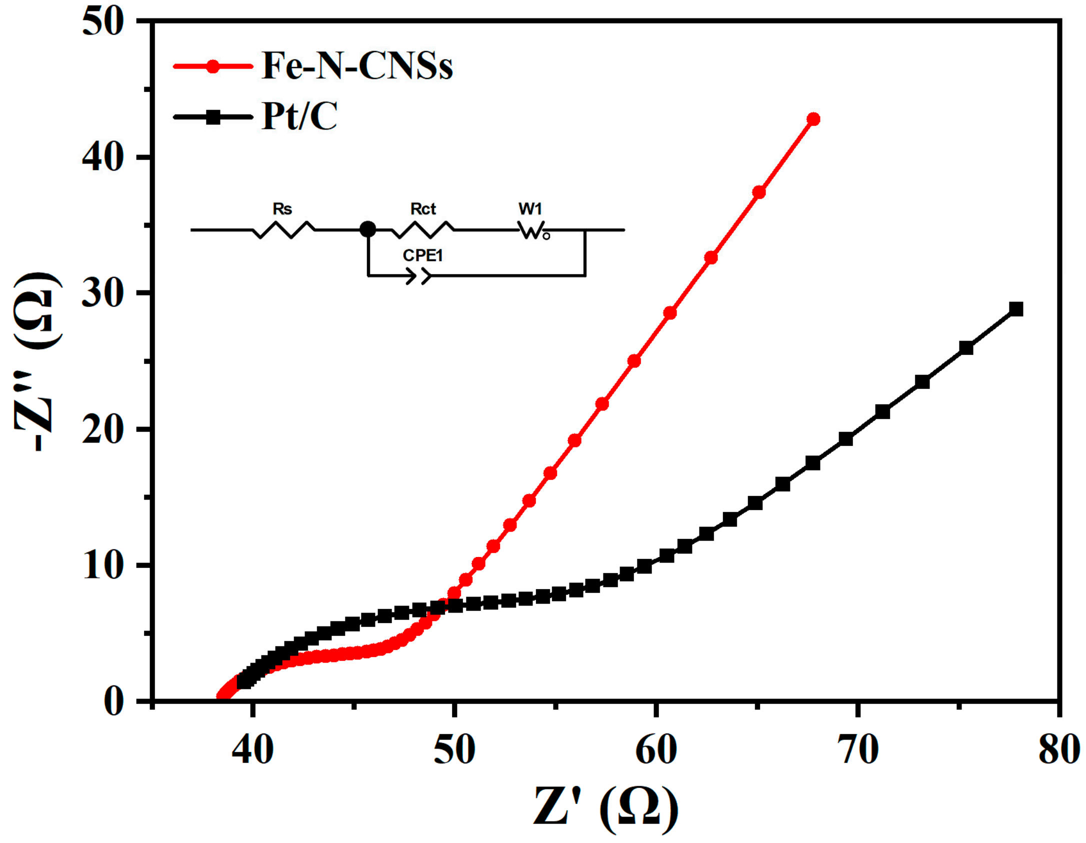

Figure **S17**. Nyquist plots of electrochemical impedance spectroscopy (EIS) for Fe-N-CNSs and Pt/C.

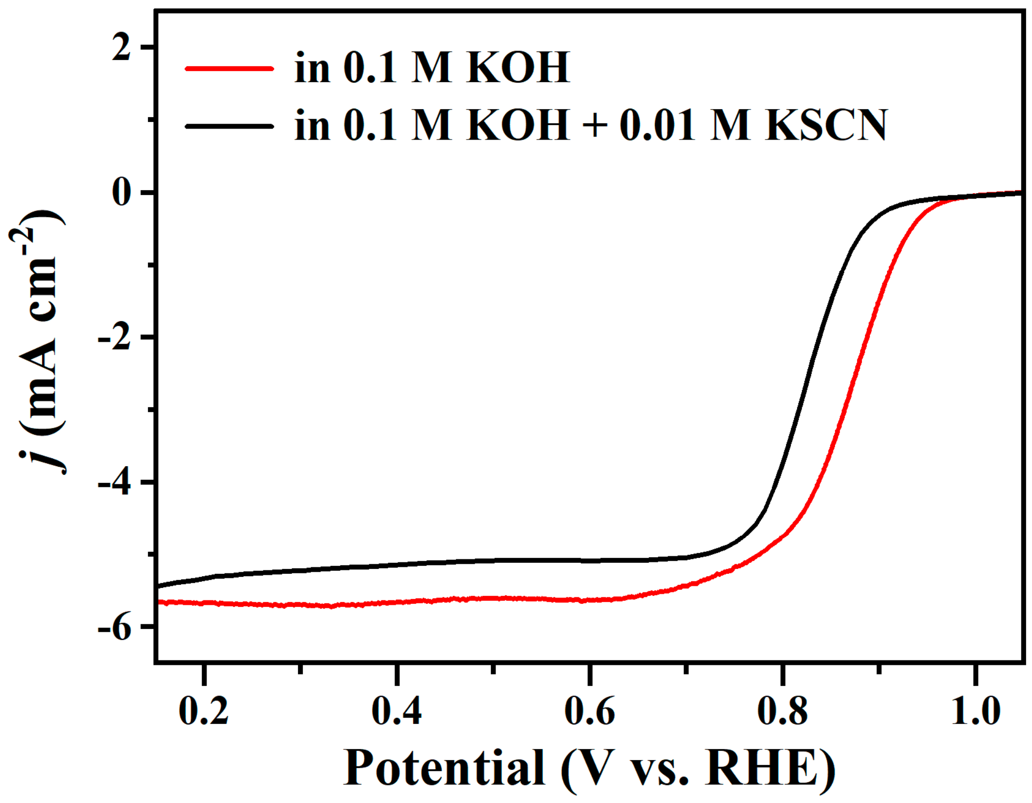

Figure **S18**. Poison experiment by KSCN.
